# A Macaque Model for the Effects of Hybridization on Body Size

**DOI:** 10.1002/ajpa.25062

**Published:** 2025-02-10

**Authors:** Laura T. Buck, David C. Katz, Rebecca Rogers Ackermann, Leslea J. Hlusko, Sree Kanthaswamy, Timothy D. Weaver

**Affiliations:** ^1^ Research Centre for Evolutionary Anthropology and Palaeoecology, School of Biological and Environmental Sciences Liverpool John Moores University Liverpool UK; ^2^ Department of Anthropology University of California Davis Davis California USA; ^3^ University of Calgary Cumming School of Medicine Calgary Canada; ^4^ Department of Archaeology University of Cape Town Cape Town South Africa; ^5^ Human Evolution Research Institute University of Cape Town Cape Town South Africa; ^6^ Centro Nacional de Investigación Sobre la Evolutión Humana Burgos Spain; ^7^ School of Mathematical and Natural Sciences Arizona State University Tempe Arizona USA

**Keywords:** dysgenesis, heterosis, hybrid, size, variation

## Abstract

**Objectives:**

Genomics research has uncovered recurrent hybridization between hominin species, yet its morphological impact remains understudied. Non‐human primate research has suggested a morphological signature of hybrid ancestry, which could be used to identify hybrids in the hominin fossil record. This pattern may include extreme size, heightened variation, and markers of developmental instability, but factors affecting these characteristics are poorly understood. Studies of non‐mammalian taxa suggest that extreme morphology is more likely in early‐generation hybrids and with a greater parental distance. To understand hybridization in hominins, therefore, we must use appropriate proxy taxa.

**Materials and Methods:**

Here, we use Chinese × Indian *Macaca mulatta* hybrids with a comparable divergence time in generations to *Homo sapiens* /Neanderthals and wide variation in admixture. Measuring limb lengths, body length, and weight, we investigate the relationship between admixture and size/variation.

**Results:**

Compared to previous work with more phylogenetically distant primate taxa and a focus on early generation hybrids, we found no evidence of a relationship between admixture and extreme large size, nor with increased size variation. Hybrids in our sample are relatively small but within the range of variation of the smaller parental taxon.

**Conclusions:**

Our results suggest that hybridization between closely related taxa, such as Neanderthals and *H. sapiens* , may lead to more subtle morphological patterns than previously anticipated. It will be necessary, however, to better understand the factors governing primate hybrid morphology before we can produce robust inferences on how hybridization has affected hominin evolution.

## Graphical abstract

Virtual measurements from CT scans of living monkeys were used to capture size and shape data on the effects of hybridization in size and variation.

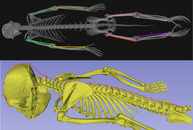

**Summary**

- Hybrids between closely related primate taxa are smaller than expected but do not fall outside the range of parental size variation.
- This may affect hybrid fitness and our ability to recognize fossil hominin hybrids from their morphology.

## 1 Introduction

Despite advances in ancient DNA (aDNA) research uncovering a complex history of admixture during human evolution (Gokcumen 2020; Gopalan et al. 2021), we still lack a thorough understanding of the morphological impact of gene flow between lineages and the ability to recognize hybrids in the fossil record (Harvati and Ackermann 2022; Warren et al. 2018). This is important because, given the conditions required for aDNA preservation, the assumptions inherent in its analysis, and the destructive nature of sampling, aDNA is a tool best used in conjunction with examination of fossil morphology. Furthermore, since selection acts upon phenotypes, it is primarily by investigating the phenotypic effects of admixture that we will better understand how hybridization may have shaped human adaptation. One such potential role is in fitting dispersing *Homo sapiens* populations to novel ecological niches during migration and global expansions (Ackermann, Mackay, and Arnold 2016; Atsumi, Lagisz, and Nakagawa 2021; Rieseberg et al. 2007), a defining feature of our lineage (Buck et al. 2019; Roberts and Stewart 2018; Wells and Stock 2007).

Studies of both captive‐bred (e.g., Ackermann, Rogers, and Cheverud 2006; Ackermann et al. 2014; Cheverud, Jacobs, and Moore 1993) and wild (e.g., Fuzessy et al. 2014; Kelaita and Cortés‐Ortiz 2013) non‐human primate species suggest that there may be a consistent signature of distinctive morphology in primate hybrids. This is reported to include size outside the range of parental variation, particularly larger size (indicative of heterosis or hybrid vigor; see Ackermann (2010) for a glossary of terms relevant to hybridization); high levels of variation; and non‐metric traits suggestive of developmental instability (Ackermann, Rogers, and Cheverud 2006; Ackermann et al. 2019). Transgressive phenotypes resulting from hybridization can contribute to adaptation by increasing variation, and large size may also be advantageous in some contexts, such as inter‐male competition for breeding. Conversely, hybridization may also be detrimental, causing what is termed dysgenesis, disrupting developmental pathways, compromising the immune system (Sage et al. 1986, c.f. Baird et al. 2012), or leading to the loss of beneficial genes. Such outcomes may be visible as small size or anomalous morphology in offspring, which can reinforce reproductive boundaries and lead to speciation (Atsumi, Lagisz, and Nakagawa 2021; Seehausen 2004).

If the non‐human primate hybrid morphological signature is robust and applies to hominins, it could be used to diagnose hybrids in the fossil record in the absence of aDNA. It has been suggested (Ackermann 2010; Ackermann et al. 2019) that some known fossil hominins, such as A.L. 198–1 (*Australopithecus afarensis*) and Neanderthals from Krapina, do display characteristics typical of hybrids, such as large size and unusual non‐metric traits. Most of the non‐human primate comparative studies on which our expectations of hybrid morphology are predicated, however, have been carried out with pairs of taxa that are relatively phylogenetically divergent compared to hybridizing hominins (see below). Many classic studies also focused on samples from the first one to three generations post‐hybridization (Ackermann, Rogers, and Cheverud 2006; Ackermann et al. 2014; Eichel and Ackermann 2016). At present, therefore, it is still unclear how phylogenetic distance and admixture proportion might affect the expression of the proposed hybrid signature and so which taxa are the most appropriate models for hominin hybridization. This hinders attempts to refine predictions of the outcomes of interbreeding events such as those between *H. sapiens* and Neanderthals.

Non‐mammalian studies suggest that extreme morphologies, including size, are more likely in early‐generation hybrids and with greater genetic divergence between parents (Stelkens and Seehausen 2009). Novel heterozygotic combinations of alleles in hybrid individuals may lead to new interactions between alleles (epistasis and dominance) (Atsumi, Lagisz, and Nakagawa 2021). The main mechanism resulting in extreme size appears to be novel combinations of alleles at antagonistic quantitative trait loci (QTLs). Fixed combinations of alleles influencing size in opposite directions cancel one another out in parental taxa but can align, following recombination in hybrid offspring, to either increase or decrease the overall size (Rieseberg et al. 2007; Stelkens and Seehausen 2009). This effect may become diluted in later generations of hybridization or with backcrossing, where the genetic input from one parent or the other may be biased due to stochastic effects or selection (Fuzessy et al. 2014). Greater phylogenetic divergence between parents is thought to lead to a more extreme hybrid morphology because greater genetic distance leads to greater numbers of QTLs fixed antagonistically (Stelkens et al. 2009). The relationship between transgressive morphology (e.g., extreme size), genetic divergence, and time since introgression remains complex and poorly understood even in heavily studied model organisms such as sunflowers and cichlids (Atsumi, Lagisz, and Nakagawa 2021; Rieseberg et al. 2007; Stelkens et al. 2009); this is even more the case for primates, both human and non‐human, for whom experimental studies are not possible.

If the morphology potentially indicative of admixture is affected by phylogenetic proximity and number of generations since the hybridization event, we need to ensure that these parameters in our non‐human primate models are appropriate for modeling hominin hybridization. *H. sapiens* and Neanderthals are sister taxa, relatively closely related, with an estimated split time of 550–765 ka (Meyer et al. 2016). This is less than the phylogenetic distance between most of the non‐human taxa that have been the focus of hybrid primate studies, for example, 1.4 Ma for the *Papio* species (Rogers et al. 2019) studied by Ackermann and her colleagues (Ackermann, 2009, 2010; Ackermann, Rogers, and Cheverud (2006); Ackermann et al. (2014), Eichel and Ackermann (2016)) and ~3 Ma (Cortés‐Ortiz et al. 2003) for the *Alouatta* species studied by Kelaita and Cortés‐Ortiz (2013). The *Callithrix* taxa studied by Fuzessy et al. (2014) have an estimated split time of ~700 ka (Malukiewicz et al. 2017), but the shorter generation times in non‐human species must also be considered, with *Callithrix* having a mean age at first conception of just 2.5 years (Tardiff et al. 2003). Furthermore, given the limited number of interbreeding events that have been modeled as the best‐supported scenario for the introgression of Neanderthal ancestry into *H. sapiens* (Bergström et al. 2021), most hybrids in the fossil record are likely to be multiple generations after initial interbreeding, the recent discovery of a first‐generation Neanderthal/Denisovan hybrid notwithstanding (Slon et al. 2018).

We use here a non‐human primate proxy that may more closely approximate the *H. sapiens* /Neanderthal phylogenetic distance and the kind of variably admixed population one might plausibly expect multiple generations after an interbreeding event. Our sample is a large, multigenerational population of admixed Chinese and Indian rhesus macaques ( *Macaca mulatta* ) housed at the California National Primate Research Center (CNPRC), University of California, Davis. These taxa have been shown to differ morphologically and genetically (Fooden 2000; Smith and Mcdonough 2005) and have a comparable number of generations since divergence to Neanderthals/ *H. sapiens* , although phenotypic divergence is much less in the macaques than the hominins (Buck et al. 2021). Unlike many non‐human hybrid studies, which focus on early generations (e.g., Ackermann, Rogers, and Cheverud 2006; Savriama et al. 2018; Warren et al. 2018), our sample has a wide range of Chinese ancestry (Table 1), which better represents the situation seen in natural hybrid zones (Kelaita and Cortés‐Ortiz 2013). We investigate the effect of hybridization on size within this sample and compare our results with non‐human primate hybrid studies of taxon pairs with greater phylogenetic distances and with different hybridity profiles.

**TABLE 1:** Full sample (*n* = 134) breakdown by sex and admixture (% Chinese ancestry). Admixture percentages were obtained from CNPRC pedigrees.

% Chinese	0	12.5	25	37.5	50	62.5	75	87.5	100
Total	19	44	23	15	7	5	7	1	13
Female	11	29	15	12	5	3	7	1	9
Male	8	15	8	3	2	2	0	0	4

## 2 Materials and Methods

The individuals in the sample were CT scanned at the CNPRC by appropriately trained staff. All procedures were approved by the Institutional Animal Care and Use Committee of the University of California, Davis (protocols #19057, #20812, and #22506). Most individuals were sedated and scanned in vivo. Although a few monkeys were scanned as cadavers, no animals were sacrificed for this study. The data used in this study (and in Buck et al. 2021) are part of an NSF‐funded project (grants #1623366, #1720128), and we are committed to making them as widely available as possible. As they become available due to natural attrition, macerated skeletal remains from monkeys involved in this study are curated in the Department of Anthropology, University of California, Davis. These are available for study following application to the Department. The CT data from this sample are freely accessible to anyone with a legitimate interest via the open repository MorphoSource.com (project name: “The rhesus macaque admixture project”). Please contact the lead author via MorphoSource to request access. For further details of the history of the sample, see Buck et al. (2021) and Kanthaswamy et al. (2009).

The sample consists of 134 (92 female, 42 male) adult *M. mulatta* (Table 1). The sex ratio of the sample is biased due to the uneven sex ratio in the CNPRC colony, which is controlled to minimize intra‐male fighting. To mitigate the potential effects of this unequal sex ratio, we have sought to keep the ratio of males to females constant across all admixture groups. Sample admixture ranges from 0% to 100% Chinese ancestry, as provided by the CNPRC pedigree, which is estimated from mating records. The pedigree‐derived eight bins of Chinese ancestry shown in Table 1 are used here to group the hybrid sample by degree of admixture. The skeletons of the sample were virtuallly segmented from full body CT scans in Avizo 9.0 Lite (Thermo Fisher Scientific 2019) using a combination of automatic and manual routines.

**FIGURE 1:**
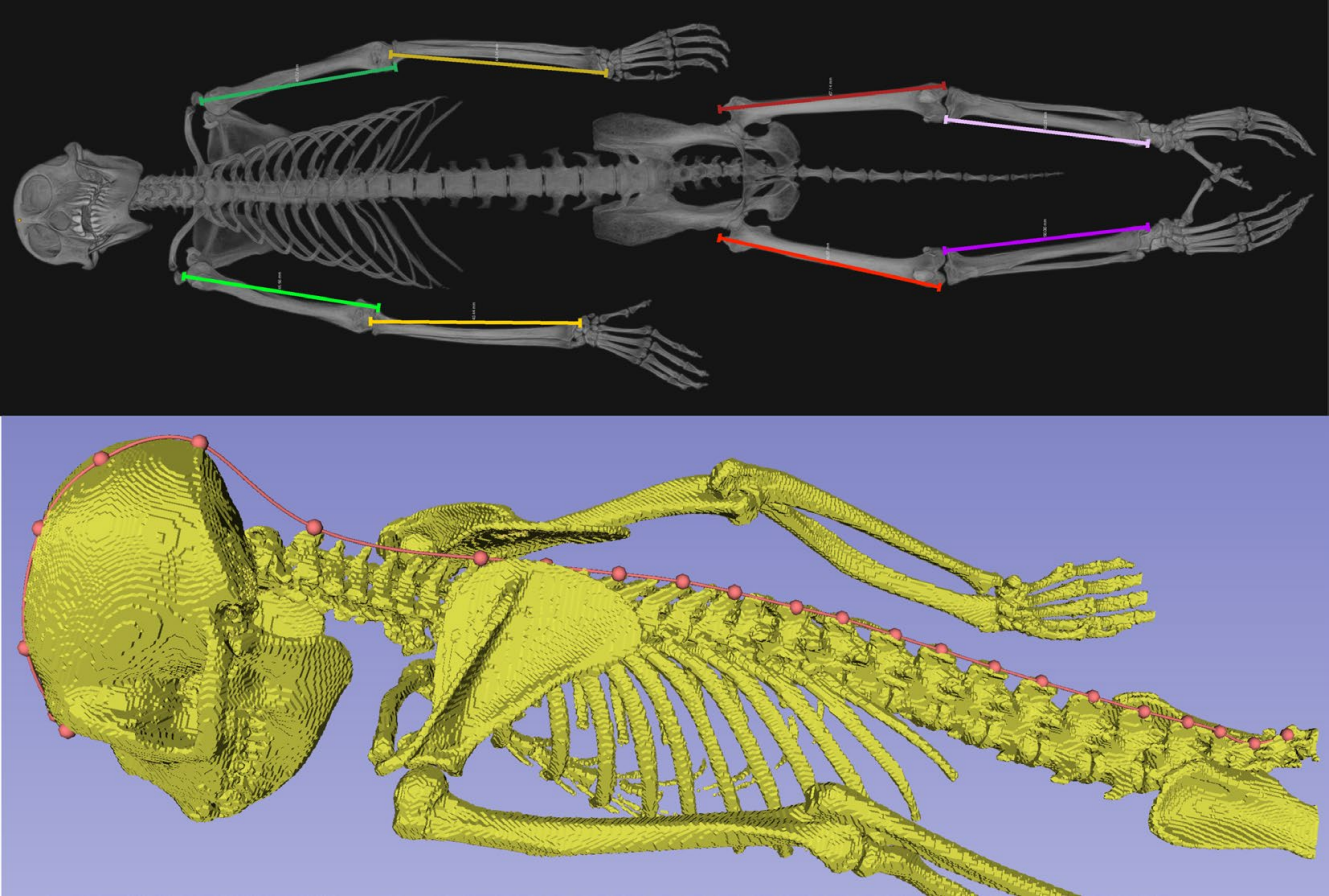
Top: Maximum limb measurements (Table 2): Green: Humeri, yellow: Radii, red: Femora, and purple: Tibiae (measured in Avizo). Bottom: Crown‐rump measurement: Glabella to notch under spinous process on S3 (measured in SlicerMorph).

Maximum lengths of limb bones (humerus, femur, radius, and tibia) were measured virtually in Avizo on the isosurfaces of segmented skeletons using the 3D linear measurement tool (Figure 1). Landmarks were placed on limb bone maxima (Table 2), and the measurements were taken between these points. Crown to rump lengths were measured in SlicerMorph (Rolfe, Davis, and Maga 2021). Surface meshes (.ply files) were first extracted from the isosurfaces of skeletal segmentations and then simplified by halving the number of triangles in Avizo to facilitate use in SlicerMorph. The meshes were exported for use in SlicerMorph, where crown‐rump lengths were measured using the Open Curve semi‐landmarking tool (Figure 1). For definitions of measurements, see Table 2. Weight in kilograms was collected at scanning by appropriately trained CNPRC staff.

**TABLE 2:** Definitions of measurements used.

Measurement	Definition
Crown to rump length	Following (Huck, Rotundo, and Fernandez‐Duque 2011). Jointed length starting at the mid‐point of line connecting mid‐points of orbits and ending at notch under the spinous process on S3. Points were included on the nuchal ridge, C2, and T1 for each individual. Additional points on the spine were placed on the spinous processes of the vertebrae, with sufficient points used to describe the shape of the spine
Maximum humeral length	Proximal‐most point on humeral head to distal‐most point on medial trochlea
Maximum radial length	Proximal‐most point on the radial head in line with the styloid process to the distal‐most point on the styloid process
Maximum femoral length	Proximal‐most point on the greater trochanter to the distal‐most point on the lateral condyle
Maximum tibial length	Proximal‐most point on the medial margin of the proximal condyle to the distal‐most point on the medial malleolus

Statistical analyses were performed in Excel (Microsoft Corporation 2018) and R (R Core Team 2021). Graphs were created in R and edited for aesthetics in Inkscape (Inkscape project 2020) where necessary. To preserve sample sizes within ancestry groups, males and females were combined. Since previous work has shown there are differences in sexual dimorphism between Chinese and Indian *M. mulatta* (Clarke and O'neil 1999), we adjusted for sex separately in each full‐bred group and in the hybrids as a single, combined group. To adjust for sex, the difference between male and female means was added to each female value. Although patterns of sexual dimorphism may differ within hybrids between admixture groups (i.e., with the amount of Chinese ancestry), as some groups do not contain males (Table 1), the sample sizes made it impossible to adjust for sex separately for each hybrid group.

To determine whether hybrid size is transgressive compared to parental taxa, we compared hybrid measurements to the means of full‐bred groups and to the expected value for each hybrid individual based on its percentage of Chinese ancestry. Expected values were calculated using the formula $\left(1-A\right){\bar{x}}_{\mathrm{Ind}}+A{\bar{x}}_{\mathrm{Chi}}$, where *A* is the percent Chinese ancestry for an individual, ${\bar{x}}_{\mathrm{Ind}}$ is the Indian mean for a measurement, and ${\bar{x}}_{\mathrm{Chi}}$ is the Chinese mean for the same measurement. By plotting actual measurements against expected measurements (Figure 3), we can determine whether hybrid individuals tend to fall above or below the parity line (the line along which the hybrid value is the same as the expected value based on full‐breds) and thus assess the case for extreme size in the hybrid sample. The theoretical rationale for the expected value is as follows: if we assume that the genetic basis of each measurement in Chinese and Indian *M. mulatta* is a very large number of genetic loci, such as is the case for height in humans (Yengo et al. 2022), then percent Chinese ancestry will be a good predictor of the percentage of the alleles at the loci underlying the measurement that derive from Chinese ancestry.^1^ So, for example, if 10,000 loci affect the measurement, and the percent Chinese ancestry for a particular individual is 80%, we expect that about 16,000 of this individual's alleles at the loci affecting the measurement will derive from Chinese ancestry (and about 4000 will derive from Indian ancestry). For a full‐bred Indian individual, in contrast, all 20,000 alleles affecting the measurement will derive from Indian ancestry. The total number of alleles (20,000) is twice the number of loci because with paired chromosomes, there are two alleles per locus. If we further assume that the effects of the alleles are equal and additive, then the effect of a particular allele on the measurement will not depend on which allele it is paired with at the locus (no dominance) or which alleles are found at other loci (no epistasis). In this case, the expected value of the measurement based on genetics alone (i.e., not accounting for non‐genetic influences on the measurement) will be the number of Chinese‐derived alleles multiplied by the average effect of a Chinese‐derived allele plus the number of Indian‐derived alleles multiplied by the average effect of an Indian‐derived allele. Therefore, the expected value for each individual is the weighted average of the full‐bred means, where the weights are individual's ancestry percentages (percent Chinese ancestry, percent Indian ancestry = 1 − % Chinese ancestry).

We used the coefficient of variance with a correction for small sample size (V*) (Sokal and Braumann 1980) to investigate variation in size between full‐bred and hybrid individuals. Independent *t*‐tests (one‐tailed) were used to test for statistically significant larger or smaller size in hybrids compared to mean full‐bred values (Cheverud, Jacobs, and Moore 1993) and between hybrid and full‐bred V*s to assess variation (Sokal and Braumann 1980).

## 3 Results

### 3.1 Size

**FIGURE 2:**
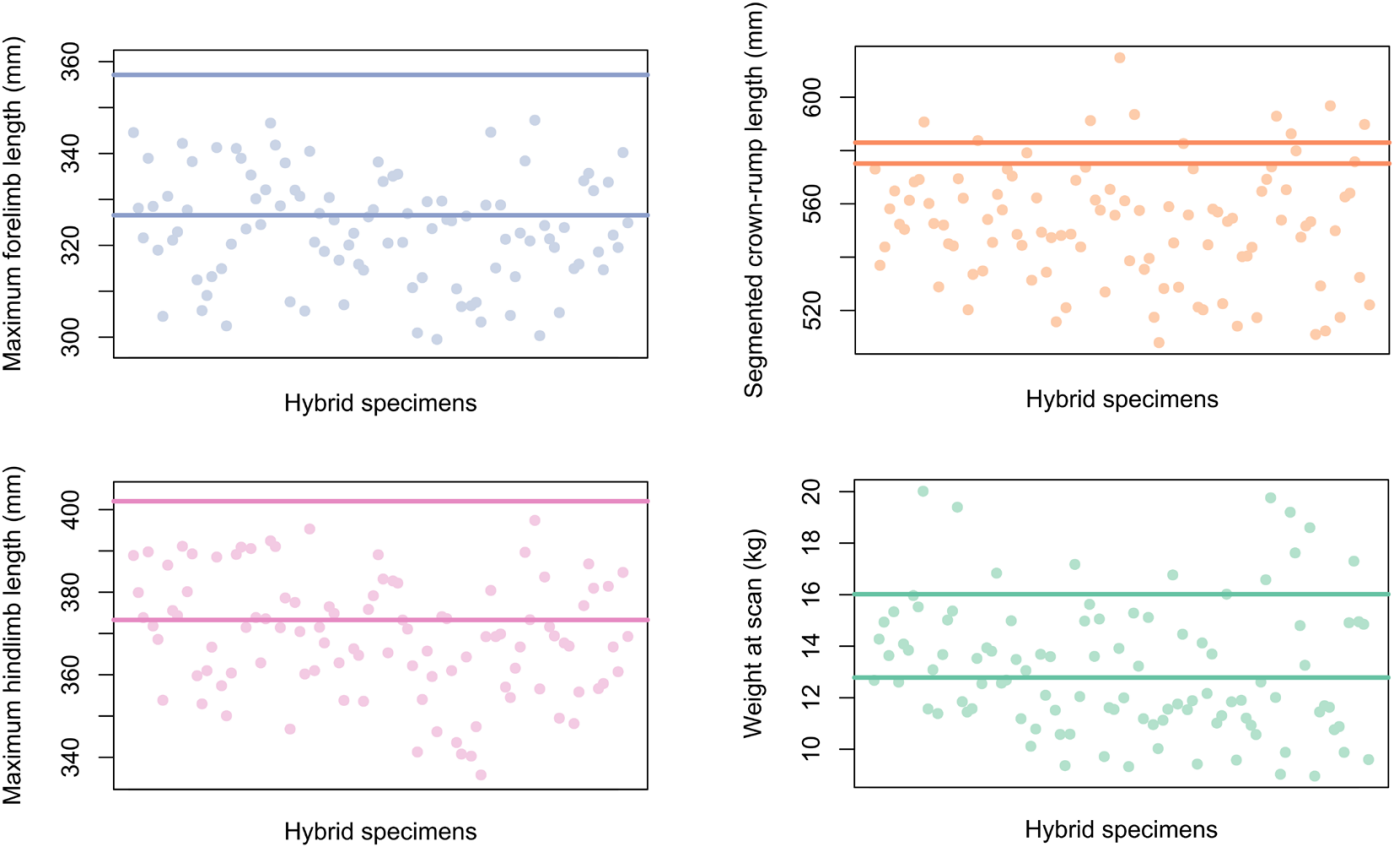
Plots showing sex‐adjusted hybrid values (points = hybrid individuals only), with parental means shown as solid, horizontal lines. Top left to bottom right: Maximum forelimb length, blue; maximum hindlimb length, pink; crown‐rump length, orange; weight at scanning, green. For limb lengths, the greater mean is the Chinese full‐bred mean, while for crown‐rump length and weight, the greater mean is the Indian full‐bred mean.

**FIGURE 3:**
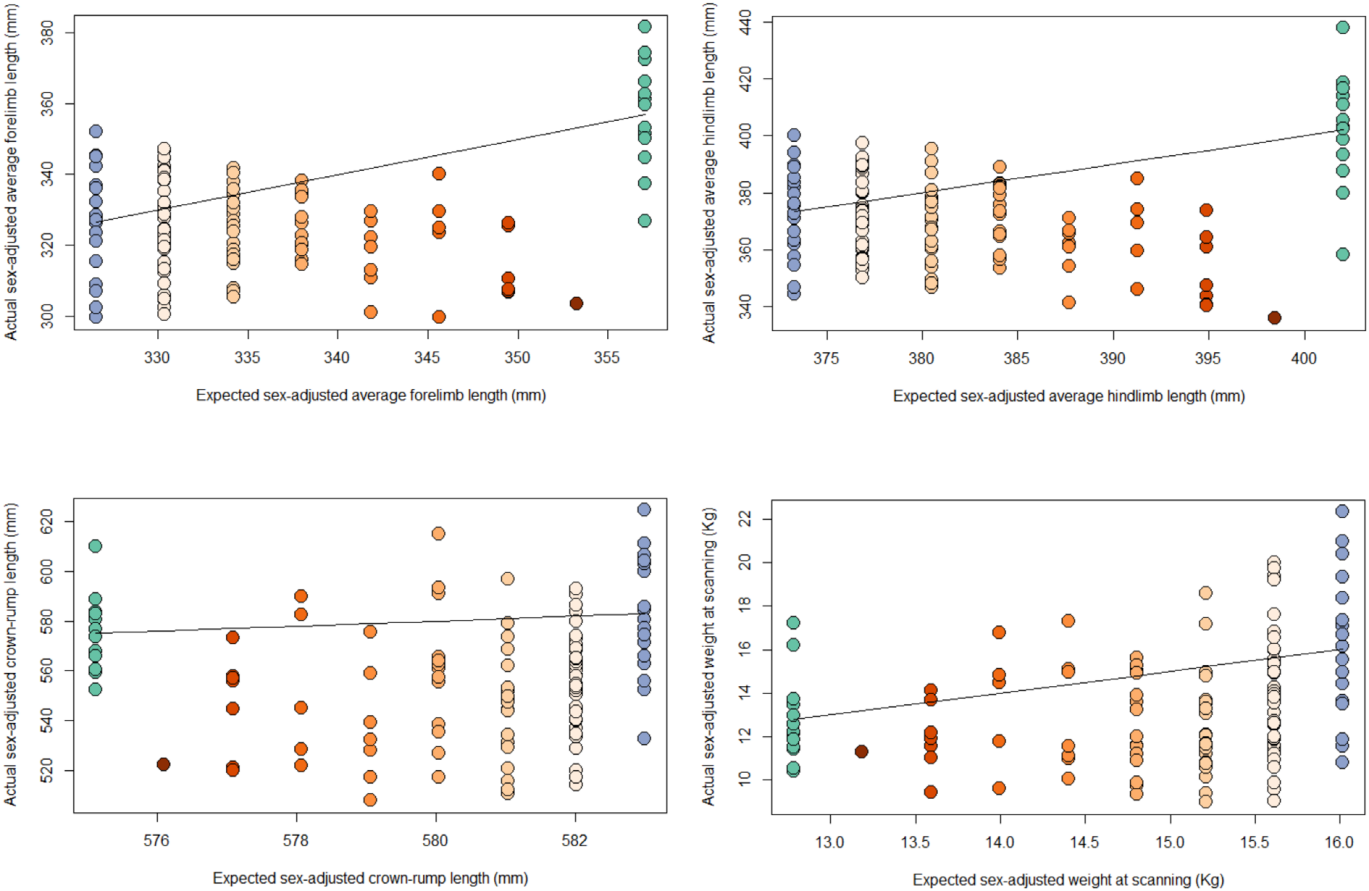
Actual versus expected sex‐adjusted values. The line represents parity between actual and expected values. Forelimb length is humerus + radius measurements, hindlimb length is femur + tibia measurements. Blue: Indian; green: Chinese. Orange: Hybrid with darker orange signifying more Chinese ancestry, from 0.125% to 0.875%.

As a group, the hybrids are consistently relatively small, being close to, or below, the smaller full‐bred mean size (Figure 2 and Table 3) for all measurements except maximum tibial length. They are significantly smaller than the greater full‐bred parent group's mean for all measurements except tibial length, but they are significantly different in size (smaller) than the smaller full‐bred parent's mean only in crown‐rump length (Table 4). There is a difference in the results for limb lengths and body size, such that there are some individuals who are larger than both parental means for crown‐rump length and weight (although hybrids as a group are smaller), but none for the limb lengths. This suggests that hybrids have disproportionately short limbs even for their generally small overall size. Full‐bred Chinese animals have longer limbs, and full‐bred Indian animals have larger bodies; thus, hybrids are closer to the full‐bred Indian mean for limb measurements and closer to the full‐bred Chinese mean for crown‐rump length and weight. Figure 3 shows how size within hybrids interacts with admixture (percentage Chinese ancestry).

**TABLE 3:** Means for measurements by group. Weight at scanning is measured in kilograms, and all other measurements are in millimeters. Forelimb is calculated as humerus plus radius and hindlimb as femur plus tibia for each individual.

Measurement	Indian mean	Chinese mean	Hybrid mean
Weight	16.02	12.78	13.08
Crown‐rump	583.00	575.13	552.19
Humerus	165.91	180.61	164.55
Femur	196.14	209.96	193.61
Radius	160.65	176.49	158.89
Tibia	177.17	192.09	175.75
Forelimb	326.56	357.10	321.89
Hindlimb	373.30	402.05	367.64

**TABLE 4:** Independent, one‐tailed *t*‐tests comparing sex‐adjusted hybrid means with (top) mid‐parental values and (bottom) greatest full‐bred means. Following Bonferroni correction, Df = 12, *Significant at *p* < 0.0083. Critical *T* value = 3.06.

	Weight	Crown‐rump	Humerus	Femur	Radius	Tibia
*Smallest full‐bred mean*						
*T*	0.48	−4.91*	−0.72	−1.32	−0.95	0.48
*p*	> 0.0083	> 0.0083	> 0.0083	> 0.0083	> 0.0083	> 0.0083
*Greatest full‐bred mean*						
*T*	−3.63*	−5.32*	−7.95*	−5.81*	−6.77*	0.31
*p*	< 0.005	< 0.005	< 0.005	< 0.005	< 0.005	> 0.0083

Figure 3 shows actual against expected values for individuals, based on the Chinese and Indian full‐bred means for each measurement and each individual's percentage of Chinese ancestry. No plot (forelimb length, hindlimb length, crown‐rump length, or weight) shows a greater number of hybrids above the parity line than below, which would be the case if there was consistently anomalous large size in hybrids. Hybrids tend to fall below the parity line, being closer to the smaller of the parental taxa for each measurement and thus generally being smaller than expected. This is particularly true in hybrids with greater percentages of Chinese ancestry in both the limb length and crown‐rump and weight graphs. This is unexpected, given that Chinese full‐breds have longer limbs than their full‐bred Indian counterparts. Furthermore, in the limb measurements, the smaller size pattern in the hybrids (particularly those with greater Chinese ancestry) is more pronounced than in the body size and weight graphs. However, as there are small sample sizes of hybrids with higher percentages of Chinese ancestry, this may be due to chance.

### 3.2 Variation in Size

**FIGURE 4:**
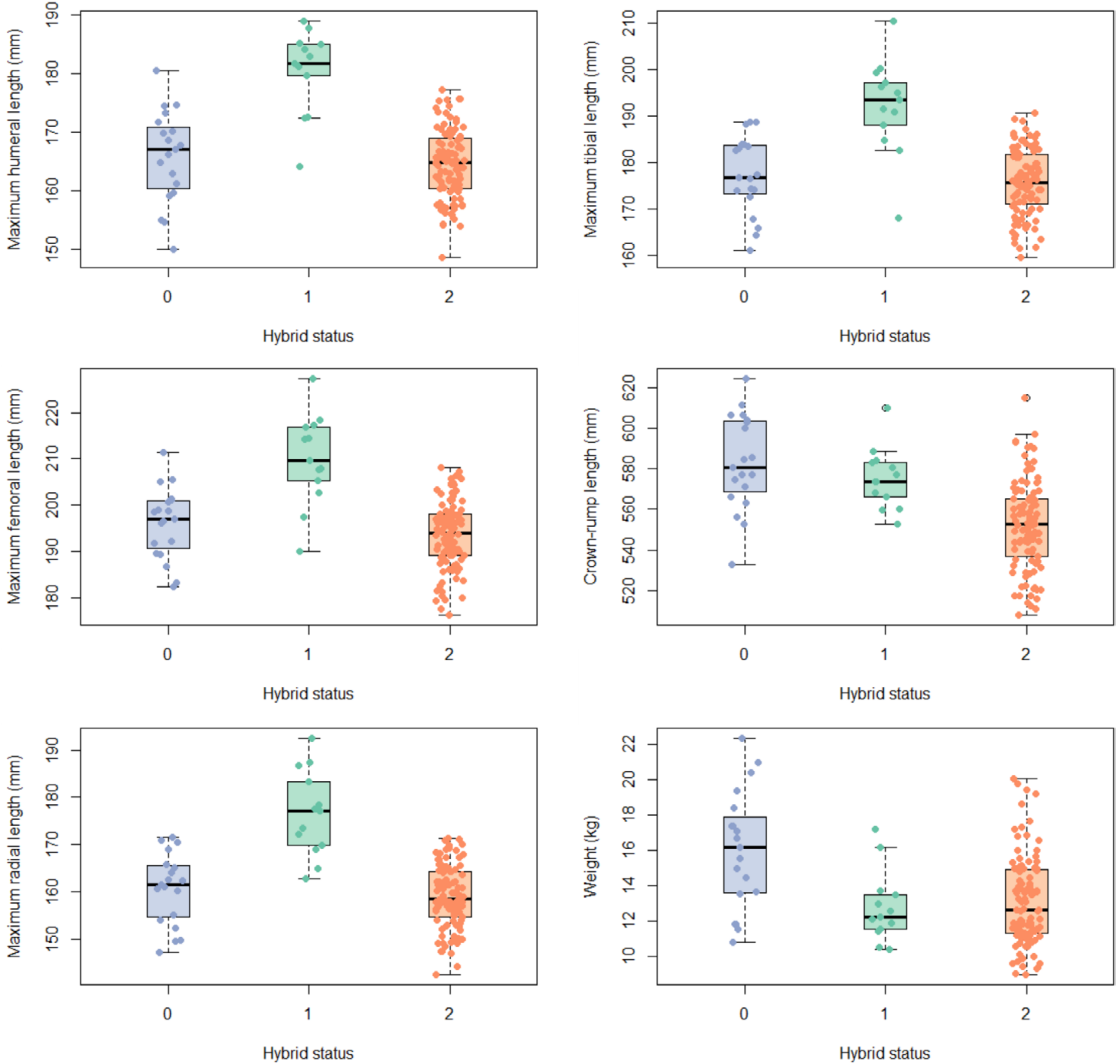
Boxplots of each of the sex‐adjusted measurements of size by hybrid status. Blue/0: Indian, green/1: Chinese, and orange/2: Hybrid.

Table 5 shows the coefficients of variation (V*) for each group for each measurement. The only variable for which variation, as measured by the coefficient of variation, is significantly different between the hybrids and either full‐bred taxon is weight, which differs between hybrids and Chinese full‐breds (Table 6). Hybrids are more variable in weight than Chinese full‐breds but not more so than Indian full‐breds. There are 102 hybrids compared to 13 full‐bred Chinese individuals and 19 full‐bred Indian individuals, so given that the hybrids are the largest sample (although note that the coefficient of variation does correct for small sample size), it is perhaps not surprising they are more variable than the Chinese sample, but it is interesting that the Indian sample is still more variable. Weight is the only instance where the hybrids are more variable than either of the full‐bred taxa (see also Figure 4).

**TABLE 5:** V* (coefficient of variation, using correction for small sample size) of hybrids and full‐breds for each measurement.

Measurement	Indian	Chinese	Hybrid
Weight	21.27	16.02	19.34
Crown‐rump	4.07	2.64	4.05
Humerus	4.79	3.93	3.59
Femur	3.97	4.76	3.79
Radius	4.76	5.24	4.25
Tibia	4.81	5.42	4.06

**TABLE 6:** Independent, one‐tailed *t*‐tests comparing V* of hybrids and full‐breds. Following Bonferroni correction, Df = 12, *Significant at *p* < 0.0083. Critical T value = 3.06.

	Weight	Crown‐rump	Humerus	Femur	Radius	Tibia
*Hybrid vs. Indian full‐bred*						
*T*	−1.99	−0.02	−0.23	−0.03	−0.09	−0.75
*p*	> 0.0083	> 0.0083	> 0.0083	> 0.0083	> 0.0083	> 0.0083
*Hybrid vs. Chinese full‐bred*						
*T*	6.03*	0.04	−0.08	−0.12	−0.15	−0.17
*p*	< 0.005	> 0.0083	> 0.0083	> 0.0083	> 0.0083	> 0.0083

## 4 Discussion

### 4.1 Size

We aim to better understand the effects of phylogenetic divergence and admixture proportion on hybrid morphology and to work toward establishing a robust non‐human primate model for hominin hybridization. Here, we collected data comparable with previous non‐human primate research in a novel, phylogenetically close, multigenerational macaque hybrid sample. To put our results into context, we first consider the relevant existing literature. Some of the earliest studies of hybrid non‐human primates were carried out in a combined captive‐bred and wild sample of saddle‐back tamarins (Cheverud, Jacobs, and Moore 1993; Kohn, Langton, and Cheverud 2001). When Cheverud and colleagues were writing, these hybrids were designated *Saguinus fuscicollis illigeri* × *S. f. lagonotus* and *S. f. illigeri* × *S. f. leucogenys*; however, the generic name has since changed to *Leontocebus* and the subspecies have been raised to species status, such that these taxa are now *Leontocebus illigeri* , *L. lagonotus* , and *L. leucogenys* (Rylands et al. 2016). The early tamarin work showed that first‐generation tamarin hybrids display extreme large size (hybrid mean greater than the mid‐parental value, which is expected size in these first‐generation hybrids) in the majority of craniofacial (Cheverud, Jacobs, and Moore 1993) and postcranial (Kohn, Langton, and Cheverud 2001) measurements. Studies of captive‐bred, known‐pedigree *Papio cynocephalus* × *P. anubis* hybrids also recorded F_1_ (first hybrid generation) and B_1_ (first back‐crossed generation) animals that were highly variable and significantly larger than their expected size based on parental means for several craniofacial traits (Ackermann, Rogers, and Cheverud 2006). This formed part of the morphological signature of heightened variation and extreme size suggested to typify hybrids, potentially including hominin hybrids (Ackermann 2010; Ackermann, Rogers, and Cheverud 2006; Ackermann, Mackay, and Arnold 2016).

Results from captive samples have been augmented by work with wild non‐human primate hybrid populations. Wild *Callithrix penicillata* × *C. geoffroyi* hybrids with unknown admixture percentages from a multigenerational, freely‐hybridizing population demonstrate a mosaic of larger and smaller size than their parental taxa for different measurements, with some individuals displaying transgressive size (Fuzessy et al. 2014). Similarly, wild crosses between *Macaca maurus* and *M. tonkeana* show transgressive large size in cranial and body length and transgressive small size in body mass (Schillaci et al. 2005). Kelaita and Cortés‐Ortiz (2013) studied a wild, multigenerational, freely‐hybridizing sample of *Alouatta pigra* × *A. palliata* and found a largely additive relationship between parental size and ancestry in many measurements, but with some extreme phenotypes outside of the range of variation for both parent species in intermediate individuals (animals with substantial ancestry from each parental taxon). A general picture emerges of marked variation and widespread extreme size (at least in some individuals) in a phylogenetically widespread sample of non‐human primate hybrids.

Here, we find that our multigenerational sample of Indian × Chinese *M. mulatta* hybrids are generally smaller than expected based on full‐bred means and ancestry, being closer in size to the smaller parent for each measurement. This transgressively small size is potentially indicative of dysgenesis. Dysgenesis is usually associated with the hybrid offspring of phylogenetically distant taxa or those adapted to markedly different environments, since it is thought to result from the breakdown of coadapted gene complexes or loss of beneficial genes (Atsumi, Lagisz, and Nakagawa 2021). It would be unexpected to find it in hybrids such as our *M. mulatta* sample, whose parental taxa split only ~162,000 years ago (Hernandez et al. 2007) and which are the end‐points of a single distribution of a relatively generalist (Fooden 2000) macaque species (albeit one that may be polyphyletic (Smith and Mcdonough 2005)). We do not know the origins of our captive‐bred sample within China and India, meaning that it is impossible to be sure exactly how different the ancestral environments of the Chinese and Indian *M. mulatta* were, but assuming them not to have inhabited substantially different niches, other potential factors affecting size in the hybrid sample should be investigated before concluding that small size results from dysgenesis.

Dominance rank is a key determiner of relationships in social non‐human primates and may affect individual life experience and fitness (e.g., Chancellor and Isbell 2008; Fourie et al. 2015; Kimock et al. 2022). Rhesus macaque society is characterized by strongly enforced independent male and female “despotic and nepotistic” dominance hierarchies (Cooper et al. 2022; Thierry et al. 2008). These hierarchies are distinctive compared to other macaque species in the general lack of reconciliatory behavior, the degree of aggression used to reinforce status, the strength of female hierarchies, and the establishment of male rank via tenure (Cooper et al. 2022). If the CNPRC colony's dominance hierarchies interacted with admixture, through rank‐mediated factors such as food access (Chancellor and Isbell 2008; Deutsch and Lee 1991), neonatal size/growth (Bowman and Lee 1995), or stress (Feng et al. 2016) these hierarchies could contribute to small hybrid size in our sample. It has been previously shown, however, that in the CNPRC colony, there is no relationship between degree of Chinese ancestry and rank, or reproductive success, in males or females (Kanthaswamy et al. 2011), meaning that it is unlikely to contribute to the small hybrid size we find. A long‐term study of wild baboon hybrids suggests that hybrid identity itself may lead to higher levels of stress independent of rank (Fourie et al. 2015). As long‐term stress is harmful for growth and health maintenance (Sapolsky 2005), it is possible a similar pattern could reduce hybrid size in our sample. Given the differences in social structure and environment, however, caution is necessary in extrapolating from wild baboons to captive rhesus macaques. In future, a deeper investigation of the social and psychosocial context of the hybrids in the CNPRC colony would be profitable to better understand hybridization in these monkeys, for example, examining social networks or mating preferences and measuring stress hormones. Macaques, like most non‐human primates, are extremely social animals and their relationships affect their lives in complex ways. This is still more true for humans (and presumably other hominin taxa), and modeling the interactions between social relationships and hybrid identity will be important for understanding the role of admixture in human evolution.

In our sample, limb lengths in particular seem small. Hybrid individuals remain closer in length to the Indian mean, even at high levels of Chinese ancestry, despite Chinese full‐breds having longer limbs on average (Figure 3). This could be an artifact of our sample composition, but it deserves further investigation. Other multigenerational non‐human primate hybrid studies, e.g., (Fuzessy et al. 2014; Schillaci et al. 2005) have also found regionally mosaic patterns in measurements of hybrid size, which may reflect regionally differential canalization in response to hybridization or variation in the relationship between genotype and phenotype within the skeleton. The results we present here for body size, particularly for limb size, seem to contrast with our previous results from analyses of hybrid pelvis morphology (Buck et al. 2021), where we found a weak but additive relationship between shape and ancestry. These analyses were of shape, not form (shape plus size), leaving the possibility that differences in size between hybrid and full‐bred pelves were masked or that the pelvis is more constrained than limb and trunk lengths due to its many competing functional requirements (Buck et al. 2010).

Previous non‐human primate research shows that extreme large size suggestive of heterosis is a frequent consequence of hybridization (see above) and one that we might have expected to see in our sample but did not for any measurement. One possible explanation may be insufficient differences in allele frequencies between our parental taxa (full‐bred Indian and Chinese *M. mulatta* ) resulting from a relatively short period of genetic isolation, i.e., these parental taxa are not phylogenetically distant enough for heterosis to arise (Cheverud, Jacobs, and Moore 1993). These two lineages of rhesus macaques split an estimated 162 ka (Hernandez et al. 2007), relatively recently compared to other taxa that showed at least some evidence of extreme large size in their hybrids: ~1.4 Ma for the *Papio* taxa (Rogers et al. 2019) studied by Ackermann and colleagues (e.g., Ackermann, Rogers, and Cheverud 2006; Ackermann et al. 2014; Eichel and Ackermann 2016), ~700 ka for the *Callithrix* species studied by Fuzessy et al. (2014), and ~3 Ma for the *Alouatta* species (Cortés‐Ortiz et al. 2003) studied by Kelaita and Cortés‐Ortiz (2013). If we do not see heterosis because our taxa split too recently, this supports the suggestion that the small size we do see in our sample does not result from dysgenesis due to the breakdown of developmental pathways and gene complexes, which would be expected to be greater with increasing phylogenetic distance between parents due to accumulated genetic divergence (Atsumi, Lagisz, and Nakagawa 2021). Several other potential reasons exist for a lack of transgressively large size in the current sample. Heterosis is most likely in the F_1_ generation due to restored heterogeneity after allele fixation in parental lineages (Fuzessy et al. 2014; Prentis et al. 2008), but there is only one F_1_ individual in our sample and few individuals with 50% Chinese ancestry. Our pooled sexes may also obscure some relationships between size and admixture, as heterosis can affect the sexes differently (Ackermann, Rogers, and Cheverud 2006; Kelaita and Cortés‐Ortiz 2013). The future inclusion of scanned but not yet segmented individuals will improve our sample sizes and enable us to examine the role of sex in mediating the effects of admixture. Additionally, our hybrid definitions, provided by the CNPRC, are based on mating records and family relationships. Since there can be error in determining hybrid identity in this way, we are currently conducting genomic analyses to better estimate admixture percentages. Our future work will compare results obtained using pedigree records and genomic estimates of Chinese ancestry.

### 4.2 Variation

Hybrids in the current sample are no more variable than full‐breds, despite the former's much larger sample size and their wide variation in percentage of Chinese ancestry. This contrasts with findings from baboons, tamarins, and howler monkeys (Ackermann, Rogers, and Cheverud 2006; Fuzessy et al. 2014; Kelaita and Cortés‐Ortiz 2013), but it does fit with our earlier results from pelvic shape analyses in these *M. mulatta* hybrids, which found no greater variation in hybrids than full‐breds (Buck et al. 2021). A study of cranial shape in hybrids between wild *Macaca fuscata* and *M. cyclopis* escaped from a Japanese zoo (Boel, Curnoe, and Hamada 2019) found similarly low levels of variation in hybrids compared to parental taxa. Boel, Curnoe, and Hamada (2019) suggested that variation in their hybrid sample was not elevated as one might expect as a result of admixture due to the relative phylogenetic closeness of the parental taxa, with *M. fuscata* and *M. cylopis* having diverged ~170 ka (Chu, Lin, and Wu 2007). This is comparable with the ~162 ka divergence estimates for the Indian and Chinese *M. mulatta* used in this study (Hernandez et al. 2007), and so we might reasonably expect a similar effect on variation in hybrids. Greater variation in hybrids is hypothesized to result from their increased genetic heterozygosity (Fuzessy et al. 2014). In the current sample, this would not be seen if substantial allele fixation in parental lineages has not had time to arise. The lack of elevated variation we see in our sample may provide additional support for the suggestion that the full‐bred taxa are not sufficiently differentiated to lead to dysgenesis, requiring another explanation for the smaller than expected size we see in the hybrids.

Comparable levels of variation between hybrids and full‐breds may also be related to the multigenerational nature of the present sample, with low numbers of early‐generation hybrids. The effect of increased heterozygosity can be reduced over the course of subsequent generations if the contribution from one parent is biased (Fuzessy et al. 2014); in our sample, the majority of individuals have greater proportions of Indian ancestry, due to the nature and history of the CNPRC population (Kanthaswamy et al. 2009). Weight variation in the current study is relatively high compared to other measurements of size. This could be related to the captive nature of our sample or potentially to co‐variation with age, which is also affected by the captive status of the monkeys. Captive primates are sometimes obese as a result, mainly, of energy dense, over‐abundant diets and perhaps the psychosocial effects of living in captivity, leading to overconsumption as a displacement activity (Pontzer 2023). In contrast, they frequently live longer than wild animals in the absence of predation, with the reduction of intraspecific competition, and given veterinary care (Tidière et al. 2016). The relationship between these factors and Chinese/Indian/hybrid ancestry could be a fruitful avenue for future investigation.

### 4.3 Inferences for Hominin Hybrids

In addition to clarifying the effects of hybridization in non‐human primates, we aim to determine which taxa may make robust proxies for modeling hybridization in hominins. Measured in terms of estimated numbers of generations, the divergence time between the Indian and Chinese *M. mulatta* used here is more similar to that of *H. sapiens* and Neanderthals than the hominin split time is to those for the *Papio*, *Callithrix*, or *Alouatta* taxa (see above). Hybrids from these latter genera and other non‐primate taxa (Ackermann et al. 2010; Warren et al. 2018) show transgressive size, especially extremely large size, and high levels of variation, leading Ackermann and colleagues to suggest that these morphological characteristics might be used to diagnose hybrids in the hominin fossil record (Ackermann 2010; Ackermann, Mackay, and Arnold 2016). Our results suggest that we might not expect to see transgressively large and extremely variable *H. sapiens* × Neanderthal hybrids. The inferences from the present study are currently unclear as to whether transgressively small size might be seen in hominin hybrids, depending on which factors lead to this result in our macaque sample, a question that needs further investigation. Even if our sample is a good proxy for what should be expected in *H. sapiens* × Neanderthal hybrids, the small size we find here is not outside of the range of parental variation. Smaller than expected size is only detectable if the expected size based on known ancestry can be calculated, which is not currently possible for most fossils due to difficulties with aDNA retrieval. It is possible that we should not expect to be able to identify individual fossil hybrids between such recently diverged taxa based only on anomalous size. The split time for Neanderthal/ *H. sapiens* divergence is not universally agreed upon, and an earlier split date for the hominin taxa (see, for example, Gómez‐Robles 2019) might lead to expectations of more transgressive phenotypes in their hybrids, more similar to that seen in other non‐human primate crosses.

Phenotypic, as well as phylogenetic, distance between parental taxa has a potential effect on hybrid morphology. The relationship between phenotypic distance between parental taxa and transgressive morphology in hybrid offspring is unclear, and there is no straightforward relationship between phylogenetic and phenotypic distance (Stelkens et al. 2009). Appropriate phenotypic distance for Neanderthal/ *H. sapiens* hybridization is harder to account for than phylogenetic distance by using non‐human primate models because it seems likely that the hominin pairs are unusually phenotypically divergent for their relatively recent split time (for discussion, see Buck et al. 2021). This unusually high phenotypic distance between Neanderthals and *H. sapiens* should be considered when determining the most appropriate models for reconstructing the effects of hominin hybridization. Ideally, information from a range of proxy taxa with different types of suitability (e.g., comparable phylogenetic divergence, or comparable phenotypic divergence) should be used to inform future models.

As mentioned above, a key determinant in the morphology of hybrids appears to be time (generations) since introgression (Prentis et al. 2008). Relative to previous primate studies and laboratory hybridization experiments, ours is a useful sample for evaluating the effects of hybridization in the fossil record due to its more naturalistic admixture profile, following generations of interbreeding and back‐crossing (Buck et al. 2021). The human data, however, caution us that following multiple generations of interbreeding between populations, there can be a mismatch between genetic and phenotypic estimates of ancestry (Kim et al. 2021). In cases where, rather than determining the phenotype for known ancestries (as in the current study), we seek to use ancestry to predict phenotype (e.g., for future fossil studies); this may mean that even patterns based on suitable non‐human primate data do not hold over many generations. This is particularly true for traits controlled by few loci (Kim et al. 2021), meaning that presumably multi‐loci traits such as size are potentially the best traits for modeling the long‐term effects of hybridization on hominin morphology.

## 5 Conclusions

In our macaque model, we find no evidence for transgressively large size, nor greater variation in hybrid size. We do find, however, that hybrid size is generally smaller than expected based on admixture percentage (although not outside the range of parental variation). This small size could result from dysgenic break down of coadapted gene complexes, but that would be unexpected in the hybrids of such closely related taxa. Other potential factors such as the influence of ancestral ecological niche and interactions between hybrid identity and stress should be investigated to clarify the causes of small hybrid size. Our results suggest that in hybrids between relatively closely related taxa, and after multiple generations of interbreeding, extreme size and morphological variation outside of the range of parental variation may not be expected.

## Author Contributions

**Laura T. Buck:** conceptualization (lead), data curation (lead), formal analysis (lead), investigation (lead), methodology (lead), project administration (equal), visualization (lead), writing – original draft (lead). **David C. Katz:** conceptualization (supporting), formal analysis (supporting), funding acquisition (equal), methodology (supporting), writing – review and editing (supporting). **Rebecca Rogers Ackermann:** conceptualization (supporting), formal analysis (supporting), funding acquisition (supporting), investigation (supporting), methodology (supporting), writing – review and editing (supporting). **Leslea J. Hlusko:** conceptualization (supporting), formal analysis (supporting), funding acquisition (supporting), investigation (supporting), methodology (supporting), writing – review and editing (supporting). **Sree Kanthaswamy:** conceptualization (supporting), formal analysis (supporting), funding acquisition (supporting), writing – review and editing (supporting). **Timothy D. Weaver:** conceptualization (equal), data curation (equal), formal analysis (supporting), funding acquisition (lead), investigation (supporting), methodology (supporting), project administration (equal), software (lead), visualization (supporting), writing – review and editing (lead).

## Conflicts of Interest

The authors declare no conflicts of interest.

## Data Availability

The data that support the findings of this study are available from the open data repository datadryad.org, under the title of this paper. Most of the CT scans used in this study are freely available to anyone with a legitimate interest via the open repository MorphoSource.com (project name: “The rhesus macaque admixture project”). In due course, the aim is to make the entire sample available.

## Endnotes

^1^
As an aside, if the genetic basis of variation in a phenotypic trait is a much smaller number of genetic loci and some generations have passed since the admixture event, then genome‐wide ancestry percentages will not be predictive of the value of the trait because the ancestry percentages at the loci underlying the trait could be quite different from the genome‐wide ancestry percentages. This insight provides part of the explanation for why, contrary to popular conception, phenotypic traits (e.g., skin color) are often uninformative about genome‐wide ancestry in humans (Kim et al. 2021; Parra et al. 2003).
